# Evaluation of Extrauterine Head Growth From 14-21 days to Discharge With Longitudinal Intergrowth-21st Charts: A New Approach to Identify Very Preterm Infants at Risk of Long-Term Neurodevelopmental Impairment

**DOI:** 10.3389/fped.2020.572930

**Published:** 2020-11-19

**Authors:** Giulia Maiocco, Giuseppe Migliaretti, Francesco Cresi, Chiara Peila, Sonia Deantoni, Beatrice Trapani, Francesca Giuliani, Enrico Bertino, Alessandra Coscia

**Affiliations:** ^1^Neonatal Care Unit, Department of Public Health and Pediatric Sciences, University of Turin, Turin, Italy; ^2^Statistical Unit, Department of Public Health and Pediatric Sciences, University of Turin, Turin, Italy

**Keywords:** extrauterine growth restriction (EUGR), anthropometric charts, neurodevelopmental outcomes of very preterm infants, Intergrowth-21st standards, extrauterine growth of head size

## Abstract

**Background:** ExtraUterine Growth Restriction (EUGR) is a common definition for in-hospital growth failure of very preterm infants. Wide heterogeneity is found in definitions and anthropometric charts used to describe EUGR.

**Aim:** We aim to compare two traditional definitions of EUGR with a newly proposed one, based on a longitudinal evaluation, that takes into account the physiological period of fluid loss after birth. We also wish to detect which definition could better predict neurodevelopmental impairment at 24 months of corrected age (CA).

**Methods:** A total of 195 infants with GA < 30 were included. EUGR was calculated both for weight and head circumference (HC). Cross-sectional EUGR was defined as measurements < 10th percentile at discharge; longitudinal EUGR was defined as Δ*z-*score < −1 between birth and discharge measurements. The new longitudinal “post-loss” EUGR definition was proposed as Δ*z-*score < −1 between measurements taken at 14–21 days of life and at discharge. Longitudinal postnatal Intergrowth-21st charts specifically built on preterm infants were used. Association with major and minor neurodevelopmental impairment at 24-month CA was assessed for each definition. *K* coefficient and ROC curve were evaluated.

**Results:** Longitudinal “post-loss” definition of EUGR for HC is the one predicting minor neurodevelopmental impairment at the multivariate analysis (OR = 3.94), and it is also associated with a worse General Quotient. The chosen cut-off (Δ*z-*score < −1) is the proper one.

**Conclusion:** HC in-hospital growth could be a more accurate tool than weight to predict neurodevelopmental outcomes and especially minor neurological impairment. Longitudinal “post-loss” definition of EUGR assessed on longitudinal charts for preterm infants could be the most appropriate definition from the methodological, clinical, and prognostic point of view.

## Introduction

In recent literature, the term ExtraUterine Growth Restriction (EUGR) is found as a common definition for growth failure that may occur early in postnatal life of very preterm infants (1), due to both inadequate nutritional intake and non-nutritional factors (i.e., morbidities, prenatal and perinatal conditions, hospital environment, etc.) (2).

EUGR is usually defined by considering weight (most times), head circumference, or length using specific anthropometric charts. There is increasing evidence to suggest that early postnatal weight and head circumference growth is related to long-term outcomes in preterm infants, especially with reference to neurodevelopment (3). Accordingly, careful evaluation of preterm infants growth in the first period of life is of crucial importance; despite this, there is no univocal agreement regarding the ideal anthropometric charts to use for assessing the in-hospital growth rates of newborns, and there is consequently no univocal agreement for defining EUGR. In the past years, a multitude of different charts have indeed been used to describe EUGR, and these have mostly been national neonatal references (4) or fetal-infant charts (5). However, these types of charts may not be the best choice to evaluate postnatal growth. Nowadays, recommendations that preterm infants should grow like a healthy fetus *in utero* have been questioned by many authors, and Villar et al. (6) have recently made a suggestion to monitor the postnatal growth of preterm infants by using the International Fetal and Newborn Growth Consortium for the 21st Century Preterm Postnatal Growth Standards.

Another critical point is represented by the wide heterogeneity of definitions; this makes the comparison of the results weak and the clinical value of EUGR difficult to assess. Substantially, it is possible to assess the following: (I) *cross-sectional definitions of EUGR*, defined as a measurement lower than a previously set percentile or *z-*score, generally at 36 or 40 weeks of postmenstrual age (PMA) or at discharge; and (II) l*ongitudinal definitions of EUGR*, defined as a Δ*z-*score lower than −1 (or −2), generally between measurements at birth and at 36 or 40 weeks of PMA or at discharge. It is evident that the two types of definitions just refer to two different and not interchangeable conditions: while *longitudinal EUGR* is the expression of a dynamic process, *cross-sectional EUGR* is statically defined and could depend more upon birth status than upon actual growth process (7, 8).

To date, birth has always been considered the initial point in defining *longitudinal EUGR*, but many authors (7, 9) starting from Cole et al. (10) have suggested taking into account the physiological loss of fluids that causes downward centile crossing during the first weeks of life of preterm infants, starting the evaluation after this period.

The most important reason for identifying EUGR is to be able to optimize nutritional intake, care, and follow-up policies for more fragile infants in order to mitigate long-term unfavorable outcomes, such as neurodevelopmental impairment or delay (7).

The aim of this present study is to compare three different definitions of EUGR to detect the most useful one as a possible predictor of neurodevelopmental impairment at 24 months of corrected age. In doing so, the new definition of *longitudinal post-loss (of fluids)* EUGR was compared to the two “traditional” *cross-sectional* and *longitudinal* ones, using the new Intergrowth-21st longitudinal Charts specifically constructed to monitor preterm infants.

## Materials and Methods

### Study Population

Infants born before 30 weeks of gestational age (GA) and assisted at the Neonatal Intensive Care Unit of University of Turin between January 1, 2006, and December 31, 2016, were retrospectively enrolled. By predefined criteria, infants were excluded if they died or were transferred to another NICU before discharge, had a diagnosis of major congenital malformation or major cerebral lesion (i.e., intraventricular hemorrhage grade III–IV or cystic periventricular leukomalacia), did not have any anthropometric measurement recorded between 14 (−3) and 21 (+3) days of life, or did not complete 24 months of corrected age follow-up. Neonatal clinical data were collected according to the Manual of Operations edited by Vermont Oxford Network (11) and extrapolated by medical records. The nutrition protocol was standardized following the criteria of adjustable fortification in use in our Unit (12).

### Anthropometric Evaluation and EUGR Definitions

Birth weight was measured within 1 h of delivery, and infant weight was then measured daily until discharge with an electronic weighting scale and recorded to the nearest 1 g. Birth crown–heel length (BL) and head circumference (HC) were measured within 1 day of delivery and then weekly and at discharge with the Harpenden neonatometer and an non-extendable measuring tape, respectively, recorded to the nearest millimeter. Measurements were taken by trained personnel according to the techniques described by Cameron (13).

EUGR was considered both for weight and HC, and three different definitions were applied: (I) *cross-sectional EUGR* was defined as a measurement < 10th percentile at discharge (occurred between 36 and 40 weeks of PMA); (II) *longitudinal EUGR* was defined as Δ*z-*score < −1 between birth and discharge measurements; and (III) *longitudinal post-loss EUGR* was defined as Δ*z-*score < −1 between measurements taken in the time interval between 14 (−3) and 21 (+3) days of life and measurements at discharge. The chosen *cross-sectional* definition (definition I) is definitely the most common definition found in Literature to describe postnatal growth restriction (7). Regarding *longitudinal* EUGR (definition II), the authors generally use a Δ*z-*score cut-off of −2 to define severe EUGR and a Δ*z-*score cut-off of −1 (as in this present study) for mild to moderate EUGR (14–16). In addition, the *longitudinal post-loss* definition of EUGR (definition III) is now proposed according to the suggestions by Fenton et al. (7) and Cole et al. (10) to evaluate postnatal growth deficits excluding the first 2–3 weeks of life, which are typically burdened by a physiological loss of fluids in very preterm infants.

All the 195 infants have been classified using EUGR definitions for weight; 134 infants had complete data for HC measurements and could be classified also on the basis of HC.

*Z-*scores values were assessed at birth according to the Intergrowth-21st Newborn Size for Very Preterm Infants (17), and for postnatal growth according to the Intergrowth-21st longitudinal Charts for Postnatal Growth of Preterm Infants (18).

### Neurodevelopmental Outcomes

Neurodevelopmental outcomes at 24 (± 6) months of corrected age were analyzed considering the neurological, ophthalmologic, and audiologic diagnoses performed by a multidisciplinary team with standardized protocols and recorded during the follow-up visits at the Neonatology Unit of the University of Turin. Minor neurodevelopmental impairment, major neurodevelopmental impairment, and General Quotient (GQ) at the *Griffiths Mental Development Scales* (19, 20) were considered.

The Griffiths Mental Development Scales Revised (Griffiths) assess the neurodevelopment from birth to 24 months, scoring performances in five domains (subscales: Locomotor, Personal-Social, Hearing and Speech, Eye and Hand Coordination, and Performance). Standardized scores for each subscale and a composite General Quotient are given. Minor neurodevelopmental impairment is a condition that limits the child in learning and adaptation. The presence of minor impairment was evaluated by a trained developing age specialist and was intended as the presence of at least one of the following: mild motor impairment, gross or fine motor coordination difficulties, muscle tone imbalance, but without definite signs of cerebral palsy (21), lower verbal expression skills than expected, or minor visual defects (i.e., strabismus, nearsightedness, or refractive defects diagnosed by a pediatric ophthalmologist). Major neurodevelopmental impairment was defined as the presence of at least one of the following: cerebral palsy [according to the Executive Committee for the Definition of Cerebral Palsy definition (22)], blindness (i.e., total or severe unilateral or bilateral visual impairment), deafness (i.e., need for unilateral or bilateral hearing systems), or a GQ < 0.70.

### Statistical Analysis

Study data are presented using the classic descriptive statistical indicators, and changes in weight and HC from birth to 14-to-21 days of life was assessed using paired t-Student. The concordance between the different EUGR definitions in identifying the same subjects was evaluated by estimating the Cohen's *K* coefficient (23); according to Landis and Koch (24), values < 0 indicate no agreement, 0–0.20 slight agreement, 0.21–0.40 fair agreement, 0.41–0.60 moderate agreement, 0.61–0.80 substantial agreement, and 0.81–1 almost perfect agreement.

The association between weight or HC *z-*scores at birth and neurodevelopmental outcomes (major and minor impairment) was evaluated by estimating crude Odd Ratio and relative 95% confident interval.

The association between the three EUGR definitions and neurodevelopmental outcomes (major and minor impairment) was assessed using Logistic regression models. Models were performed separately for EUGR for weight and for HC. Morbidities that occur during hospital stay and are unequivocally associated both to early postnatal growth and to neurodevelopmental outcomes were considered as confounding factors. They are the following: bronchopulmonary dysplasia (BPD), i.e., request for oxygen support at 36 weeks of PMA, necrotising enterocolitis (NEC) requiring surgery, and retinopathy of the premature (ROP) requiring surgery (25, 26). The diagnoses of morbidities were performed according to the Manual of Operations edited by Vermont Oxford Network (11). In addition, birth weight z-scores (for the EUGR for weight analysis), birth HC *z-*scores (for the EUGR for HC analysis), sex, and weeks of GA were also considered as adjustment factors.

ROC curve (27) allowed us to evaluate the predictivity of weight and HC (measured in *z-*scores) on the neurodevelopmental impairment. Results are shown by estimating the area under the curve (AUC) and the relative 95% confidence interval. Youden's index was used to define the best cut-off (28); the likelihood ratio positive (LH+) with relative 95% confidence interval was estimated to validate it (29).

All the statistics were performed with SAS® Statistics Software and Statistical Package for Social Sciences 25.0 (SPSS, SPSS Inc., Chicago, IL).

## Results

**Figure 1:**
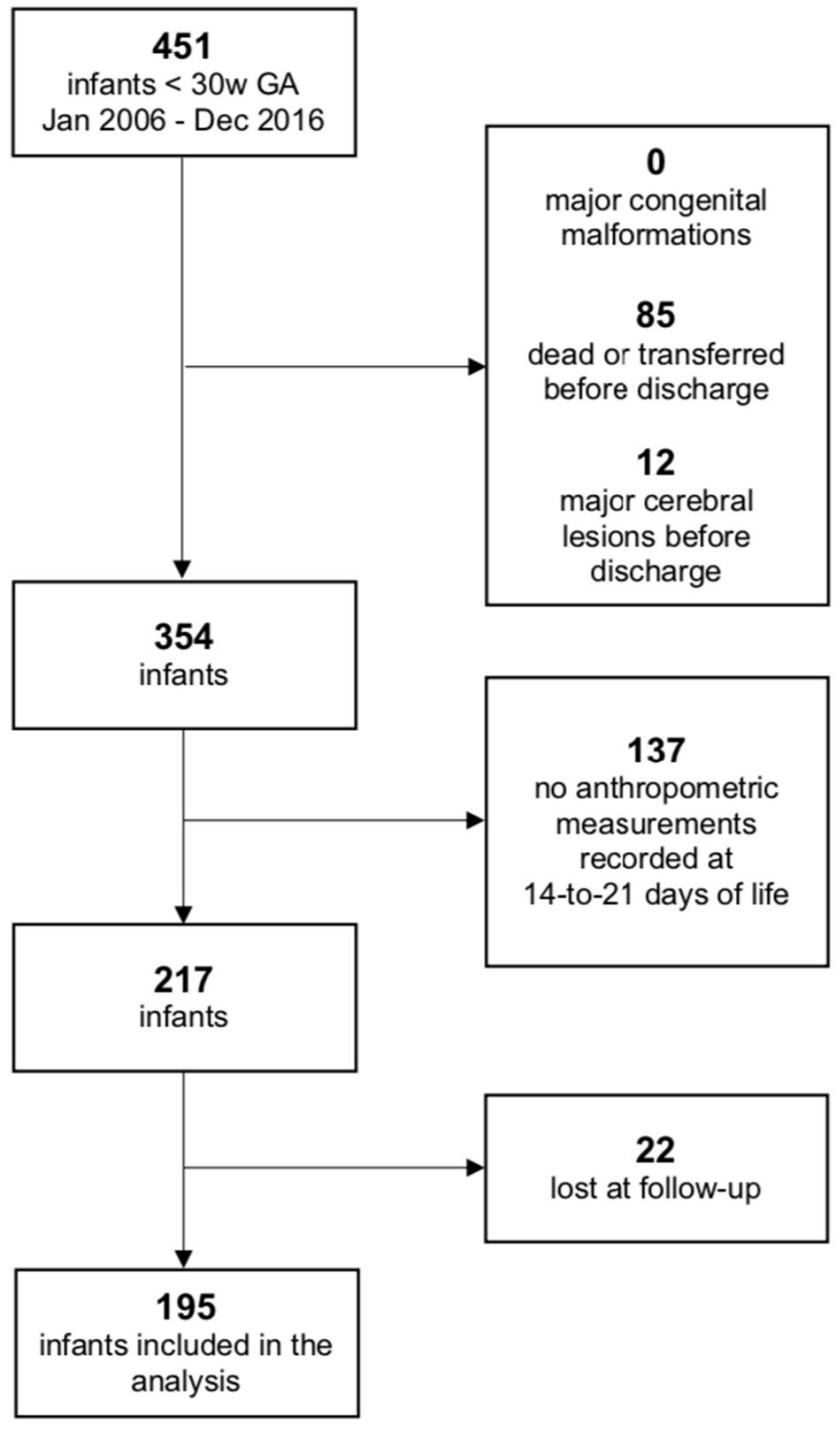
Flowchart of inclusion and exclusion criteria.

During the defined study period, a total of 451 infants born before 30 weeks of GA were enrolled. A total of 97 infants were excluded due to major cerebral lesions or early death/transfer and 159 because of lack of anthropometric or follow-up information, as shown in Figure 1; 195 infants were finally included in the analysis. Comparison between the included population and the 159 fortuitously excluded infants has shown no significant differences regarding the main clinical and anthropometric characteristics recorded during hospital stay (Supplementary Tables 1, 2). Clinical and anthropometric features of the study population and postnatal growth data are presented in Table 1. Infants who are classified as SGA (Small for Gestational Age, i.e., birth weight lower than the 10th percentile) or have a birth HC measurement lower than the 10th percentile are more than the expected 10% (27.7 and 20.0%, respectively) due to the higher probability of very preterm infants of being born IntraUterine Growth Restricted (IUGR) in our high risk pregnancies specialized Center. Both for weight and HC, the percentage of infants defined as EUGR is much lower, according to the *longitudinal post-loss definition*, than the *cross-sectional* or *longitudinal* ones. Indeed, a significant loss in *z-*scores (*p* < 0.001) is evident for both weight and HC measurements taken between birth and 14–21 days of life, which is as expected (Table 2).

**Table 1:** Clinical and anthropometric features of the study population (*N* = 195).

Female, *n* (%)	102 (52.3%)
Multiple gestation, *n* (%)	63 (32.3%)
Weeks of GA at birth, median (range)	28 (24–29)
Birthweight grams, mean (SD)	959 (262)
Birthweight *z-*scores, mean (SD)	−0.687 (1.210)
SGA, *n* (%)	54 (27.7%)
Weight at discharge grams, mean (SD)	1,843 (497)
Weight at discharge *z-*scores, mean (SD)	−2.057 (1.418)
Cross-sectional EUGR for weight, *n* (%)	137 (70.3%)
SGA and cross-sectional EUGR for weight, *n* (%)	54 (100.0%)
Longitudinal EUGR for weight, *n* (%)	128 (65.6%)
SGA and longitudinal EUGR for weight, *n* (%)	28 (51.9%)
Longitudinal post-loss EUGR for weight, *n* (%)	74 (37.9%)
SGA and longitudinal post-loss EUGR for weight, *n* (%)	19 (35.2%)
Birth HC centimeters, mean (SD)	25.1 (1.9)
Birth HC *z*-scores, mean (SD)	−0.505 (0.878)
Birth HC < 10th percentile, n (%)	39 (20.0%)
HC at discharge centimeters, mean (SD)	31.0 (2.4)
HC at discharge *z*-scores, mean (SD)	−1.713 (1.565)
Cross-sectional EUGR for HC, *n* (%)^*^	74 (55.2%)
Birth HC < 10th percentile at birth and cross-sectional EUGR for HC, *n* (%)^*^	23 (92.0%)
Longitudinal EUGR for HC, *n* (%)^*^	70 (52.2%)
Birth HC < 10th percentile at birth and longitudinal EUGR for HC, *n* (%)^*^	15 (60.0%)
Longitudinal post-loss EUGR for HC, *n* (%)^*^	17 (12.7%)
Birth HC < 10th percentile at birth and longitudinal post-loss EUGR for HC, *n* (%)^*^	4 (16.0%)
Morbidities during hospital stay, *n* (%)^**^	54 (32.1%)
BPD, *n* (%)^***^	46 (27.9%)
Surgical NEC, *n* (%)	2 (1.0%)
Surgical ROP, *n* (%)	14 (7.2%)
Weeks of GA at discharge, median (range)	36 (35–40)

* *computed on 134 infants*.

** *computed on 168 infants*.

*** *computed on 165 infants*.

**Table 2:** Weight and HC *z-*scores differences between birth and 14–21 days of life.

**Weight** ***z-*****scores (N = 195)**			**HC** ***z-*****scores (N = 134)**		
**Birth mean (SD) [95% C.I.]**	**14–21 days mean (SD) [95% C.I.]**	**Δ mean (SD) [95% C.I.]**	**Birth mean (SD) [95% C.I.]**	**14–21 days mean (SD) [95% C.I.]**	**Δ mean (SD) [95% C.I.]**
−0.69 (1.21) [−0.86; −0.52]	−1.15 (1.26) [−1.33; −0.97]	**−0.46 (0.57)** **[−0.54; −0.38]**	−0.51 (0.88) [−0.65; −0.36]	−1.68 (1.04) [−1.85; −1.50]	**−1.22 (0.71)** **[−1.34; −1.10]**

*Bold values represent statistically significant results*.

Concordance among subjects defined as EUGR for weight using the three different definitions is low: *K* = 0.33 (95% C.I. from 0.18 to 0.48) between the *cross-sectional* and the *longitudinal* definition; *K* = 0.30 (95% C.I. from 0.17 to 0.42) between the *cross-sectional* and the *longitudinal post-loss* definition; and *K* = 0.41 (95% C.I. from 0.29 to 0.53) between the *longitudinal* and the *longitudinal post-loss* definition. Regarding the EUGR definitions for HC, a discreet concordance is found only between the traditional *cross-sectional* and *longitudinal* definitions (*K* = 0.65, 95% C.I. from 0.54 to 0.76), whereas *longitudinal post-loss definition* is discordant with both the *cross-sectional* and the *longitudinal* definition (*K* = 0.16, 95% C.I. from 0.01 to 0.31 and *K* = 0.21, 95% C.I. from 0.04 to 0.37, respectively). For this reason, the association between EUGR and neurological outcomes was assessed separately for each of the proposed definitions. Prevalence of major and minor neurodevelopmental impairment at 24 months of corrected age in the study population is shown in Table 3. The small percentage of infants affected by major impairment (5.3%) makes the analysis on major impairment difficult to perform.

**Table 3:** Neurodevelopmental outcomes of very preterm infants (*N* = 195) at 24 months of corrected age.

Major neurodevelopmental impairment, *n* (%)^*^	**10 (5.3%)**
Cerebral palsy, *n* (%)^**^	8 (4.1%)
Blindness, *n* (%)	0 (0.0%)
Deafness, *n* (%)	0 (0.0%)
General Quotient < 0.70, *n* (%)^*^	2 (1.1%)
**Minor neurodevelopmental impairment, *n* (%)**	**61 (31.3%)**
Motor or verbal expression deficit, *n* (%)	53 (27.2%)
Minor visual defects, *n* (%)	17 (8.7%)

* *computed on 188 infants*.

** *computed on 193 infants*.

**Figure 2:**
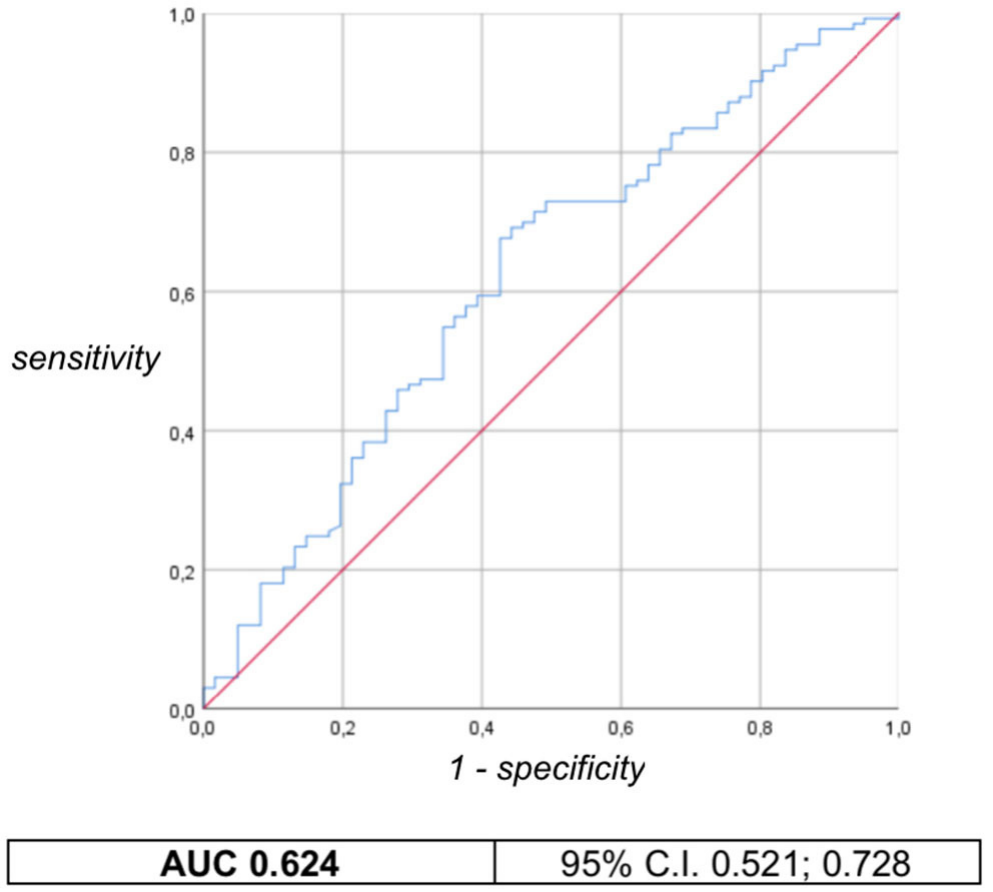
*Longitudinal post-loss* definition of EUGR for HC: ROC curve and AUC with 95% confident interval.

The association between weight and HC *z-*scores at birth and minor developmental impairment is statistically significant, as shown in Table 4. This result is supported by literature and demands the use of birth weight and HC *z-*scores as confounding variables for the evaluation of minor impairment in our study population. Results of the analysis performed to assess the possible association between different EUGR definitions and neurological outcomes are presented in Table 5. At the univariate analysis, *cross-sectional EUGR* for weight and *longitudinal post-loss EUGR* for HC definitions are both associated with minor impairment. After adjustment for the confounding variables, only the *longitudinal post-loss* definition maintains a statistically significant predictive value. Difference in GQ between the two populations (EUGR infants vs. not-EUGR) was thus assessed only for the *longitudinal post-loss* definition regarding HC: EUGR infants have a statistically significant worse GQ, as shown in Table 6. The estimated ROC curve confirms the validity of *longitudinal post-loss* definition of EUGR for HC as a discriminating measure (Figure 2). A deeper analysis based on Youden's index also highlights that a value of Δ*z-*score of −1 seems to be a good cut-off in predicting minor neurodevelopmental impairment, confirmed by a LH+ = 2.82 (95% C.I. from 1.93 to 3.72). This means that subjects defined as *longitudinal post-loss EUGR* are almost three times more likely to develop minor impairment than not to develop it.

**Table 4:** Evaluation of possible association between weight and HC *z-*scores at birth and major and minor neurodevelopmental impairment at 24 months of corrected age.

***Z-*scores**	**Major impairment**	**Minor impairment**
	**Crude OR [95% C.I.]**	**Crude OR [95% C.I.]**
Birth Weight	0.92 [0.55; 1.54]	**0.74** **[0.58; 0.95]**
Birth HC	0.78 [0.55; 1.11]	**0.77** **[0.65; 0.92]**

*Univariate analysis with crude Odd Ratio (OR). Bold values represent statistically significant results*.

**Table 5:** Evaluation of possible association between EUGR definitions and major and minor neurodevelopment impairment at 24 months of corrected age.

**Trait**	**EUGR definition**	**Major impairment**	**Minor impairment**	
		**Crude OR [95% C.I.]**	**Crude OR [95% C.I.]**	**Adjusted OR [95% C.I.]**
WEIGHT^*^	Cross-sectional EUGR	3.92 [0.49; 31.71]	**2.13** **[1.03; 4.41]**	1.11 [0.43; 2.84]
	Longitudinal EUGR	1.25 [0.31; 4.99]	0.81 [0.43; 1.52]	0.70 [0.34; 1.46]
	Longitudinal post-loss EUGR	2.49 [0.68; 9.13]	1.47 [0.79; 2.73]	0.93 [0.42; 2.10]
HC^**^	Cross-sectional EUGR	2.68 [0.55; 13.01]	1.72 [0.88; 3.35]	0.83 [0.35; 1.97]
	Longitudinal EUGR	2.99 [0.62; 14.51]	1.02 [0.53; 1.93]	0.69 [0.33; 1.45]
	Longitudinal post-loss EUGR	1.67 [0.18; 15.92]	**3.35** **[1.18; 9.51]**	**3.94** **[1.19; 13.03]**

* *analysis computed on 168 infants*.

** *analysis computed on 112 infants*.

*Crude and adjusted Odd Ratio (OR) for morbidities during hospital stay, birth weight z-scores (EUGR for weight), birth HC z-scores (EUGR for HC), sex, and GA. Bold values represent statistically significant results*.

**Table 6:** Differences in General Quotient between longitudinal post-loss EUGR and not-EUGR populations regarding HC.

**General Quotient**	**EUGR (*N* = 17)**	**Not-EUGR (*N* = 111)**	*p*-value^*^
Median	0.896	0.975	
Mean (SD) [95% C.I.]	0.93 (0.08) [0.888; 0.966]	0.97 (0.10) [0.951; 0.988]	**0.024**

* *nonparametric Mann–Whitney test was performed. Bold values represent statistically significant results*.

## Discussion

Although EUGR has been widely used in recent literature to describe postnatal growth failure of preterm infants, there is still no consensus about its definition, and this makes it difficult to compare the results of different studies and to assign EUGR its correct clinical and prognostic value. Many definitions have been proposed by authors in the last 20 years (8, 30), and the ones chosen for this study are the most used respectively for the *cross-sectional* and *longitudinal* category (cf. methods). Moreover, to our knowledge, this is the first time the *longitudinal post-loss* definition of EUGR is applied despite it having been proposed since 2014 by the group led by Cole (10). Very preterm infants experience a physiological loss of fluids immediately after birth, which leads to a downward centile crossing during the first weeks of life. For this reason, the infant's target centile should not be assessed at birth but at 14–21 days of life, which is when weight gain has steadied. This statement is consistent with our population's results, where a statistically significant difference between birth and 14–21 days of life weight measurements is shown. Moreover, the same occurs for HC measurements, likely as the result of the same loss of water. This allows us to apply the *longitudinal post-loss* definition of EUGR both to weight and HC.

Methodological heterogeneity among studies on EUGR is also found in a range of different anthropometric charts used to assess *z-*scores values: most of them are national neonatal references (4) or fetal–infant charts (5). However, from an auxological point of view, these types of charts do not represent the best choice to evaluate postnatal growth, as they refer to cross-sectional extracted birth data.

To overcome this drawback, in our study postnatal *z-*scores were assessed according to the Intergrowth-21st longitudinal Charts for Postnatal Growth of Preterm Infants (18) published in 2015, which are the first standards constructed by following-up a population of healthy preterm infants worldwide.

In this regard, a main difference between the traditional *longitudinal birth-to-discharge* definition of EUGR and our proposed *post-loss one* is that the first definition demands we combine *z-*scores derived from two distinct charts (cross-sectional neonatal chart for auxological evaluation at birth, and longitudinal postnatal chart for evaluation at discharge), whereas the *longitudinal post-loss EUGR* is calculated on a single, postnatal longitudinal chart.

In contrast, *cross-sectional definition* of EUGR only require one-time measurement assessments, but this is not the best way to define postnatal growth restriction since it does not reflect the dynamic process of growth. In our population, this is demonstrated by the fact that 100% of SGA newborns (having weight measurement under the 10th percentile at birth) are EUGR, according to the *cross-sectional* definition, even if they have not experienced a *z-*scores fall; for HC, the results are similar.

It is well-known that very preterm infants are at risk of neurological impairment during childhood (31, 32), and there is a growing interest in studying the association between postnatal growth deficit and neurodevelopmental outcomes. Major neurodevelopmental impairment is a cause of great concern due to its lifelong implications, but thanks to the advances in care system and technology it is now rare. Moreover, the primary cause of major neurological impairment during childhood are major neurological lesions (i.e., intraventricular hemorrhage grade III–IV or cystic periventricular leukomalacia), conditions we considered as exclusion criteria. In our study population, a limited number of infants presented with major neurodevelopmental impairment at 24 months of the corrected age (10 infants), and this did not allow us to perform a statistically valid analysis.

On the contrary, minor neurodevelopmental impairment is far more frequent, and our study focused on this one. The relationship between EUGR for weight and mild neurological outcomes is controversial. The traditional *cross-sectional* definition of EUGR seems not to be associated to adverse neurodevelopment on the whole (7). Chien et al. (33) find that *cross-sectional EUGR* for weight is a severity-dependent predictor of low mental developmental index at 24 months of corrected age, but the association is statistically significant just when EUGR is defined as a *z-*score lower than −3 at discharge, which is when infants show very bad general conditions. Shah et al. (34) and Zozaya et al. (35) both compare the *cross-sectional* definition of EUGR to the *longitudinal one* and show that *longitudinal EUGR* has the best predictive value for neurodevelopmental impairment. However, none of them use specific preterm longitudinal charts to calculate *z-*scores.

Considering weight growth in our study population, univariate analysis shows a statistically significant association between *cross-sectional EUGR* and minor impairment, whereas no association is found for the other proposed definitions. When correcting for the confounding variables, none of the variables predominate in the association with outcomes, and *cross-sectional EUGR* predictive value loses its statistic significance. This means that weight at discharge (*cross-sectional EUGR)* is itself the resulting effect of many perinatal variables and conditions, but it is not directly associated with neurodevelopmental impairment: weight is a rough indicator of growth.

By contrast, HC is more related to brain growth (36), thus it could be more accurate in predicting neurological outcomes. Ehrenkranz et al. (37) observe that weight and HC growth velocities during hospital stay of extremely low birth weight infants (ELBWi) are both associated with neurodevelopment, but the association is more evident for HC growth. Neubauer et al. (38, 39) assess that poor postnatal HC growth during the first months of life of very preterm infants is associated with worse neurodevelopmental outcome at 24 months of corrected age and at 5 years. Leppanen et al. (40) suggest that, for predicting low cognition outcome in SGA very preterm infants, the most significant time period for HC growth is around term age. Sicard et al. (41) define suboptimal head growth in a *longitudinal* way, and they observe an association with non-optimal neurodevelopmental outcome at 24 months of corrected age. Furthermore, small HC *z-*score at birth (lower than −2) and suboptimal head growth have a synergic effect in increasing the risk for neurocognitive impairment.

In this context, our findings underline the predictive value of HC, and show that *longitudinal post-loss EUGR* for HC is significantly associated with minor neurodevelopmental impairment at 24 months of CA after the correction for the confounding variables.

Consistently with the results shown above, these infants also have a lower GQ. Normally, GQ is impaired by major motor deficit, often associated with serious brain injury. However, in our study population there is only a limited number of infants presenting major neurodevelopmental impairment; in this case, therefore, a lower GQ mainly reflects a minor developmental impairment and is probably related to worse cognitive abilities.

Neither the *cross-sectional* nor the traditional *longitudinal* definition of EUGR for HC are associated with outcomes in our study population: this observation supports the fact that the *longitudinal post-loss* way to describe EUGR may be the most suitable in predicting neurodevelopmental prognosis.

Our study has some limitations: it has been conducted on a relatively small number of patients with single-center design, and retrospective enrollment of patients led to the exclusion of 159 subjects. HC postnatal missing data caused a further reduction of the population considered to calculate EUGR for HC (from 195 to 134 infants).

However, the analysis performed to compare the fortuitously excluded, and the included population has shown no significant differences. Follow-up drop out was <15%, which is an indicator of validity of long-term outcomes assessment. The included population is restricted to a narrow range of GA, and all the subjects received uniform feeding and care practices; moreover, anthropometric data, and neurological assessment have been computed by the same trained operators.

Our study could be considered as a preliminary study to assess the value of *longitudinal post-loss* EUGR definition in clinical practice. Further research could apply our proposed definition to a greater number of multicentric populations, performing prospective enrollment of patients with *ad hoc* data collection.

## Conclusion

HC growth during hospital stay could be a more accurate indicator to predict neurodevelopment than weight growth and should be carefully monitored by Neonatologists. Postnatal measurements of preterm infants should not be plotted on neonatal or fetal–infant charts but on longitudinal charts specifically built for preterm infants, such as the Intergrowth-21st longitudinal Charts for Postnatal Growth of Preterm Infants. EUGR for HC could be a useful tool to identify infants with a higher risk of long-term adverse outcomes, but a consensus regarding its definition is needed. Our proposed *longitudinal post-loss* definition of EUGR could be appropriate from a methodological and clinical point of view to describe in-hospital postnatal growth failure in very preterm infants, and it has been demonstrated to be associated with minor neurodevelopmental impairment and worse GQ at 24 months of corrected age.

## Data Availability Statement

The data analyzed in this study is subject to the following licenses/restrictions: to protect proprietary information, dataset access is restricted to operators of the Neonatal Care Unit of the University of Turin. Anyway, anonymous data could be available upon request. Request to access these datasets should be directed to the corresponding author.

## Ethics Statement

The studies involving human participants were reviewed and approved by Comitato Etico Interaziendale—AOU Città della Salute e della Scienza di Torino, Turin, Italy. Written informed consent to participate in this study was provided by the participants' legal guardian/next of kin.

## Author Contributions

GMa and GMi equally contributed to the work. GMa, FC, and AC conceptualized and designed the study. GMa, CP, and BT collected the data. GMi conceptualized and conducted the statistical analysis. GMa, EB, and SD drafted the initial manuscript. FC, CP, BT, FG, and AC reviewed and revised the manuscript. EB and AC supervised the work. All authors approved the final manuscript as submitted and agree to be accountable for all aspects of the work.

## Conflict of Interest

The authors declare that the research was conducted in the absence of any commercial or financial relationships that could be construed as a potential conflict of interest.
