# Supplementary material for: Evaluation of Extrauterine Head Growth From 14-21 days to Discharge With Longitudinal Intergrowth-21st Charts: A New Approach to Identify Very Preterm Infants at Risk of Long-Term Neurodevelopmental Impairment
Source: Front Pediatr. 2020 Nov 19;8:572930. doi: 10.3389/fped.2020.572930 (PMC7711073; doi:10.3389/fped.2020.572930)
Supplement: Supplementary file 1 [file Data_Sheet_1.docx]

Supplementary Material

**Supplementary Table 1.** **Comparison of categorical neonatal variables between the included and the fortuitously excluded population. Chi-square test (p-value adjusted with Bonferroni correction for multiple comparisons).**

|  | INCLUDED POPULATION  (N = 195) | EXCLUDED POPULATION  (N = 159) | p-value |
| --- | --- | --- | --- |
| Female, n (%) | 102 (52.3%) | 84 (52.8%) | 0.93 |
| SGA, n (%) | 54 (27.7%) | 32 (20.1%) | 0.57 |
| Birth HC < 10^th^ percentile, n (%) | 39 (20.0%) | 28 (17.7%)* | 0.81 |
| BPD, n (%) | 46 (27.9%)** | 40 (30.0%)*** | 0.92 |
| Surgical NEC, n (%) | 2 (1.0%) | 2 (1.3%) | 0.94 |
| Surgical ROP, n (%) | 14 (7.2%) | 19 (11.9%) | 0.58 |

*computed on 158 infants.

**computed on 165 infants.

***computed on 138 infants.

**Supplementary Table 2. Comparison of quantitative neonatal variables between the included and the fortuitously excluded population. Mann-Whitney test (p-value adjusted with Bonferroni correction for multiple comparisons).**

|  | INCLUDED POPULATION  (N = 195) | EXCLUDED POPULATION  (N = 159) | p-value |
| --- | --- | --- | --- |
| Weeks of GA, mean (SD) | 27.7 (1.43) | 27.2 (1.78) | 0.19 |
| Birthweight grams, mean (SD) | 959 (262) | 948 (252) | 0.78 |
| Birthweight z-scores, mean (SD) | –0.687 (1.210) | –0.410 (1.14) | 0.29 |
| Birth HC centimeters, mean (SD) | 25.1 (1.9) | 24.8 (2.10)* | 0.59 |
| Birth HC z-scores, mean (SD) | –0.505 (0.878) | –0.416 (0.943)* | 0.57 |

***** computed on 158 infants.
